# TGFβ-facilitated optic fissure fusion and the role of bone morphogenetic protein antagonism

**DOI:** 10.1098/rsob.170134

**Published:** 2018-03-28

**Authors:** Max D. Knickmeyer, Juan L. Mateo, Priska Eckert, Eleni Roussa, Belal Rahhal, Aimee Zuniga, Kerstin Krieglstein, Joachim Wittbrodt, Stephan Heermann

**Affiliations:** 1 Department of Molecular Embryology, Institute of Anatomy and Cell Biology, Faculty of Medicine, University of Freiburg, Freiburg D-79104, Germany; 2 Faculty of Biology, University of Freiburg, Schaenzlestrasse 1, Freiburg D-79104, Germany; 3 Departamento de Informática, Universidad de Oviedo, Jesús Arias de Velasco, Oviedo 33005, Spain; 4 Developmental Genetics, University of Basel Medical School, Basel CH-4058, Switzerland; 5 Centre for Organismal Studies, Heidelberg D-69120, Germany

**Keywords:** optic fissure fusion, coloboma, TGFβ, BMP, ECM

## Abstract

The optic fissure is a transient gap in the developing vertebrate eye, which must be closed as development proceeds. A persisting optic fissure, coloboma, is a major cause for blindness in children. Although many genes have been linked to coloboma, the process of optic fissure fusion is still little appreciated, especially on a molecular level. We identified a coloboma in mice with a targeted inactivation of transforming growth factor β2 (TGFβ2). Notably, here the optic fissure margins must have touched, however failed to fuse. Transcriptomic analyses indicated an effect on remodelling of the extracellular matrix (ECM) as an underlying mechanism. TGFβ signalling is well known for its effect on ECM remodelling, but it is at the same time often inhibited by bone morphogenetic protein (BMP) signalling. Notably, we also identified two BMP antagonists among the downregulated genes. For further functional analyses we made use of zebrafish, in which we found TGFβ ligands expressed in the developing eye, and the ligand binding receptor in the optic fissure margins where we also found active TGFβ signalling and, notably, also gremlin 2b (*grem2b*) and follistatin a (*fsta*), homologues of the regulated BMP antagonists. We hypothesized that TGFβ is locally inducing expression of BMP antagonists within the margins to relieve the inhibition from its regulatory capacity regarding ECM remodelling. We tested our hypothesis and found that induced BMP expression is sufficient to inhibit optic fissure fusion, resulting in coloboma. Our findings can likely be applied also to other fusion processes, especially when TGFβ signalling or BMP antagonism is involved, as in fusion processes during orofacial development.

## 1. Introduction

The optic fissure is a transient gap in the developing vertebrate eye. It is an entry route used by cells of the periocular mesenchyme and embryonic vasculature. However, it is necessary that the fissure is closed as development proceeds. A persisting fissure is termed coloboma. A coloboma can affect vision severely and is a frequent cause for blindness in children [1]. Many genes and signalling pathways have been linked to coloboma [2–11], resulting in a growing gene coloboma network [12,13]. Coloboma is frequently part of a multi-organ syndrome, like CHARGE syndrome or renal coloboma syndrome, linked to Chd7 and Pax2, respectively [14–18]. Notably, the morphology of coloboma phenotypes is highly variable. Alterations in some signalling pathways (e.g. Wnt, Hippo) result in vast extended coloboma [4,7], likely originating from early morphogenetic defects. In this context, we recently linked a precocious arrest of a bilateral neuroretinal flow during optic cup formation to an extended coloboma [11]. There, we found that a locally expressed antagonist to bone morphogenetic proteins (BMPs) was necessary to maintain the tissue flow. Importantly, such massive coloboma phenotypes are morphologically different from coloboma resulting from a hampered fusion process of the optic fissure margins. The pathomechanisms behind such phenotypes, however, are largely elusive, and so is the underlying physiological process. This process is not understood on a structural and especially on a molecular level.

Preceding the fusion, the prospective neuroretina and retinal pigmented epithelium (RPE) share a basement membrane within the optic fissure margin. In order to facilitate the fusion of the margins, the structure of the epithelial margins must be rearranged somehow. This was shown to affect cell–cell connections [19] and likely affects the extracellular matrix (ECM) as well. Concerning cell–cell connections it should be noted that mutants for N-cadherin and α-catenin showed coloboma phenotypes [20,21]. However, it remains unclear at which point during margin disassembly, fusion or consecutive reassembly of the neuroretina and RPE these factors play a role. Although it was shown *per se* that the dissolution of the basement membrane occurs [19] and is a prerequisite for fusion [22], the effector molecules for the structural remodelling and epithelial disassembly, which facilitate the fusion process, are largely elusive.

Transforming growth factor β (TGFβ) signalling is well known to induce changes to the ECM and, furthermore, to trigger epithelial to mesenchymal transition in various processes during development and disease [23–28]. Notably, TGFβ-regulated changes to the ECM are frequently inhibited by BMP signalling [29–32].

Here we addressed the role of TGFβ and BMP in optic fissure fusion, making use of mouse (*Mus musculus*) and zebrafish (*Danio rerio*).

Our findings show that TGFβ signalling is acting pro-fusion upon the optic fissure margins. We identified a coloboma phenotype in the TGFβ2 knockout (KO) mouse, with TGFβ ligands expressed in the zebrafish eye, and the ligand binding receptor expressed within the fissure where we also found active TGFβ signalling. We identified two TGFβ-dependent BMP signalling antagonists in mouse, and homologues of these we found expressed within the optic fissure margins in zebrafish. Notably, in zebrafish, both TGFβ signalling inhibition and forced BMP expression during fissure fusion are sufficient to prevent optic fissure fusion, resulting in coloboma.

Based on our data we propose that TGFβ signalling is locally inducing BMP antagonists to relieve a BMP-induced inhibition on ECM remodelling, eventually allowing TGFβ signalling to act pro-fusion.

## 2. Results

### 2.1. Loss of TGFβ2 results in coloboma

**Figure 1.**
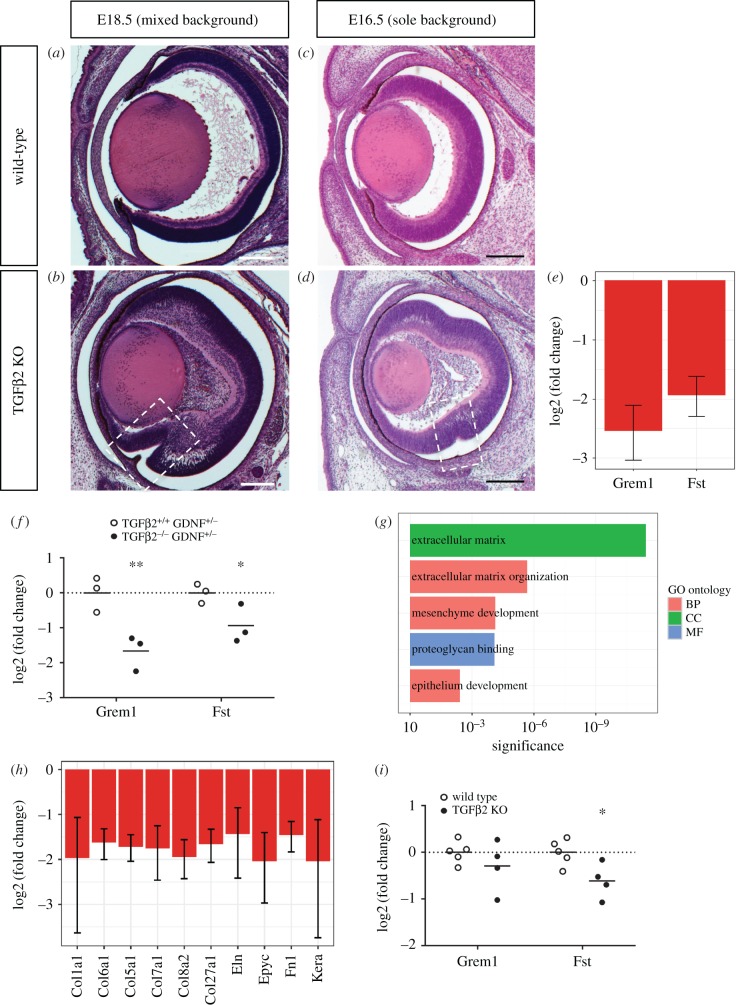
Loss of TGFβ2 ligand results in coloboma. (*a*,*b*) Frontal sections (E18.5, H&E) of (*b*) TGFβ2 KO and (*a*) wild-type mouse embryos from mixed genetic background. Note the persisting optic fissure (boxed in *b*), scale bars 200 µm. (*c*,*d*) Frontal sections (E16.5, H&E) of (*d*) TGFβ2 KO and (*c*) wild-type mouse embryos from a sole genetic background, mild coloboma phenotype boxed in (*d*), Scale bars 200 µm. (*e*) Expression analysis of gremlin and follistatin, decrease in TGFβ2 KO (TGFβ2/GDNF KO) as represented by the log2(fold change). Error bars represent the 95% confidence interval. Corrected *p*-values of control gene expression compared to KO for Grem1 and Fst, 5.5 × 10^−3^ and 1.2 × 10^−3^, respectively. (*f*) Expression analysis of gremlin and follistatin by quantitative PCR, decrease in TGFβ2 KO (TGFβ2^−/−,^ GDNF^+/−^) compared to controls (TGFβ2^+/+^, GDNF^+/−^) as represented by the log2(fold change) of individual samples; *n* = 3, horizontal bars represent the arithmetic mean. *p*-values for Grem1 and Fst, 7.8 × 10^−3^ and 0.029, respectively. (*g*) Selected terms enriched in the set of downregulated genes in TGFβ2/GDNF KO microarray based on gProfiler analysis. BP, biological process; CC, cellular component; MF, molecular function. (*h*) Expression analysis of several ECM-related genes. Error bars represent the 95% confidence interval. (*i*) Expression analysis of gremlin and follistatin by quantitative PCR, differential expression in TGFβ2 KO (sole genetic background) compared to wild-type as represented by the log2(fold change) of individual samples; *n* = 5, one KO sample was excluded as an outlier. Horizontal bars represent the arithmetic mean. *p*-Values for Grem1 and Fst, 0.312 and 0.027, respectively.

In the mouse genome, three TGFβ isoforms are encoded (TGFβ1, 2 and 3). Targeted inactivation of TGFβ2 results in several phenotypes, also affecting the eye [33], e.g. a remaining primary vitreous, a Peters anomaly like phenotype and an altered neuroretinal layering. In addition to these phenotypes, we identified a persistent optic fissure in TGFβ2 mutant embryos (figure 1*b*, electronic supplementary material, figure S1A, B, figure 1*a* as control). TGBβ2-dependent coloboma was first observed in TGFβ2/GDNF double mutants ([34], Rahhal & Heermann 2009, unpublished observations, electronic supplementary material, figure S1C) and subsequently in TGFβ2 single mutants, derived from the same breeding background (this study, figure 1*b*, electronic supplementary material figure S1A, B), but not in GDNF single mutants. Furthermore, no phenotype affecting eye development was described in the three distinct GDNF mutant mice [35–37], although GDNF expression was documented in the developing eye [38]. Notably, we found marked, severe coloboma phenotypes in both TGFβ2 KO (figure 1*b*, electronic supplementary material, figure S1A, B) as well as in TGFβ2/GDNF double KO conditions (electronic supplementary material, figure S1C). Overall, the optic fissure margins in our colobomatous embryos must have been in close proximity to each other, but ultimately failed to fuse and instead grew inwards towards the lens (figure 1*b*, electronic supplementary material, figure S1A, B, showing different sections of individual eyes). Since we found such coloboma phenotypes in both TGFβ2 and TGFβ2/GDNF mutants, but not in GDNF mutants, we found it likely that the phenotype was resulting from a TGFβ2 loss. A sensitizing role of GDNF in this scenario, however, cannot be ruled out.

So far, we based the analyses on TGFβ2 single mutants from a mixed breeding background [34] (figure 1*b*, see *a* as control). We next asked whether this breeding background could have an effect on the analyses. We additionally addressed TGFβ2 mutants derived from a sole background. Notably, while the overall coloboma phenotype was variable in intensity in the TGFβ2 single mutants from a mixed breeding background (e.g. figure 1*b*, electronic supplementary material, figure S1A, B), we could only detect very subtle forms of coloboma in TGFβ2 single mutants from a sole background (figure 1*d*, figure 1*c* as control). This suggests that the breeding background has an effect on the coloboma phenotype.

### 2.2. TGFβ signalling affects expression of bone morphogenetic protein antagonists and extracellular matrix remodelling

Many genes have been linked to coloboma [12,13]. However, optic fissure fusion is still not well understood on the structural and the molecular level. For optic fissure fusion to occur, the ECM has to be remodelled intensively. TGFβ signalling is well known for its control of ECM remodelling in various processes [23,28,39]. We thus addressed the potential transcriptional ECM regulation during optic fissure fusion using our coloboma model. We quantified the levels of mRNAs from E13.5 embryonic eyes using Agilent microarrays. To this end we compared RNA harvested from eyes of wild-type embryos and TGFβ2/GDNF double mutant embryos, in which the coloboma phenotype was assigned to TGFβ2 function.

Notably, we found the expression of two BMP antagonists, follistatin (Fst) and gremlin (Grem)1, was downregulated in the coloboma model (figure 1*e*). We validated the regulation of these genes by quantitative PCR comparing RNA from eyes of TGFβ^+/+^ GDNF^+/−^ and TGFβ2^−/−^ GDNF^+/−^ embryos (figure 1*f*).

Furthermore, we processed the obtained microarray data, focusing on significantly downregulated genes. Performing bioinformatics analysis, we found as most prominent terms ECM, ECM organization, mesenchyme development, epithelium development or proteoglycan binding (figure 1*g*). We found lower expression of collagen genes Col1a1, Col6a1, Col5a1, Col7a1, Col8a2 and Col27a1 in combination with lower levels of elastin (Eln) and epiphycan (Epyc) (figure 1*h*), pointing towards lower levels of fibrillogenesis in the extracellular space.

In addition, we isolated RNA from eyes dissected from TGFβ2 single mutants from a sole background at E12.5. The shift to E12.5 was necessary due to the fact that in the sole background condition the optic fissure fusion was occurring earlier than in the mixed background condition. Focusing on the interaction between TGFβ and BMP signalling, we quantified the expression of the two BMP antagonists. We found a significant downregulation of Fst, but no significant downregulation of Grem1 (figure 1*i*).

### 2.3. TGFβ ligands, the ligand binding receptor and TGFβ signalling in zebrafish optic cups

**Figure 2.**
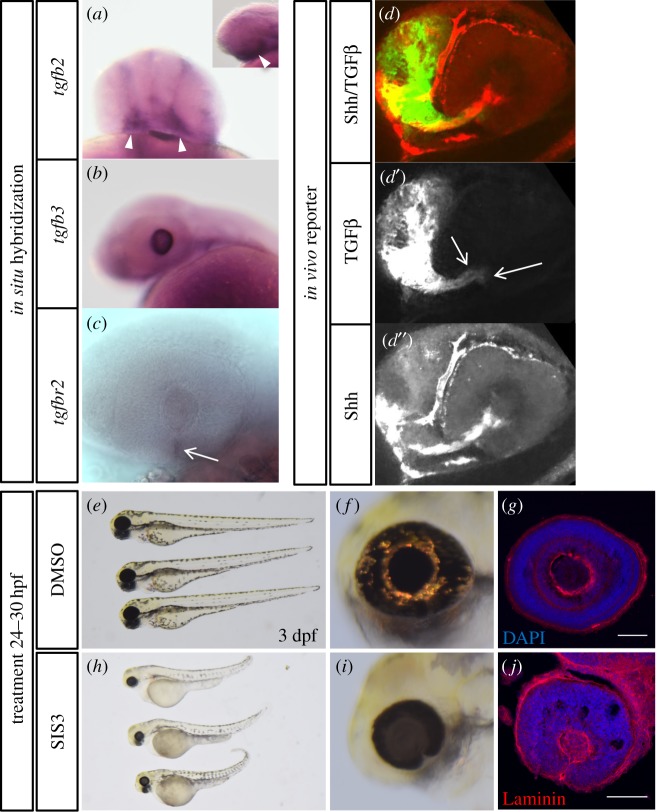
TGFβ in the zebrafish eye. (*a*) Whole-mount *in situ* hybridization (WMISH) of *tgfb2* (30 hpf), frontal view. Small image shows a lateral view. Note expression in periocular tissue (arrowheads). (*b*) WMISH of *tgfb3* (30 hpf), lateral view. Note expression in the developing lens. (*c*) WMISH of *tgfbr2* (30 hpf), lateral view. Note expression in the optic fissure (arrow). (*d*) *In vivo* signalling for TGFβ (green) and Shh (red) for orientation, split into TGFβ (*d*′) and Shh (*d*″), at 24 hpf. Active TGFβ signalling in the optic fissure margins (*d*′, arrows). (*e*–*j*) Embryos (3 dpf) treated with DMSO (*e*–*g*) or specific inhibitor of Smad3 (SIS3, *h*–*j*) from 24 to 30 hpf. Sagittal sections through eyes of (*g*) DMSO and (*j*) SIS3-treated embryos at 3 dpf, stained with DAPI and anti-Laminin. SIS3-treated embryos show a persisting optic fissure with persisting basal lamina and absence of retinal layering.

Next, we wanted to further address the functional role of TGFβ signalling for optic fissure fusion and the interplay with BMP signalling. To this end we switched to zebrafish (*D. rerio*). To ensure that a switch of model system to zebrafish is feasible, we investigated the expression of TGFβ ligands and the TGFβ ligand binding receptor during zebrafish eye development. We found *tgfb2* expressed in periocular tissue (figure 2*a*) whereas *tgfb3* was expressed in the developing lens (figure 2*b*). The ligand binding receptor *tgfbr2b* we found expressed at the site of the optic fissure (figure 2*c*). To assess the dynamics of activated TGFβ signalling *in vivo* during zebrafish development, we established a transcriptional TGFβ sensor in a transgenic zebrafish line. The reporter system is based on Smads, the canonical transcription factors transducing TGFβ signalling [40]. We used repetitive Smad binding elements (SBEs) from the human plasminogen activator inhibitor (PAI) (electronic supplementary material, figure S2A). Such a reporter has been intensively used for years as a luciferase assay to assess the amount and activity of TGFβ in cell culture [41] and in mice [42]. We then established a transgenic zebrafish line. Activated TGFβ signalling can be observed during development, e.g. in the forebrain region as well as in the distal tail (electronic supplementary material, figure S2B). We validated the functionality of this reporter line employing the established TGFβ signalling inhibitor SB431542 [43,44] (electronic supplementary material, figure S2C). Next, we wanted to know whether TGFβ signalling was active in the optic fissure margins. We employed the TGFβ signalling reporter line in combination with a reporter for Shh signalling (analogous to [45]) to relate the fissure to the optic stalk and performed imaging. We found the TGFβ signalling reporter active within the optic fissure margins (figure 2*d*,*d*′, see Shh-reporter activity for orientation (*d*″)). Taken together, our data indicate that TGFβ signalling is indeed active in the optic fissure margins of the zebrafish. Furthermore, we wanted to test if TGFβ signalling is functionally involved in optic fissure fusion in zebrafish. In order to do this, we applied the compound inhibitor specific inhibitor of Smad3 (SIS3) to wild-type embryos during the onset of optic fissure fusion from 24 to 30 hpf. SIS3 prevents phosphorylation of Smad3 [46], which, together with Smad2, is the main transcription factor in the canonical TGFβ signalling pathway. Treatment with SIS3 yielded smaller embryos at 3 dpf (figure 2*h*,*i*; see *e*,*f* as control). The tail extension as well as the brain development seemed affected, matching the expression domains we observed in the TGFβ reporter embryos (compare electronic supplementary material, figure S1B, C). Most importantly, the treated embryos had coloboma with a persisting basal lamina at 3 dpf in conjunction with reduced eye size (figure 2*i*,*j*).

### 2.4. TGFβ mediated bone morphogenetic protein antagonism during optic fissure closure

**Figure 3.**
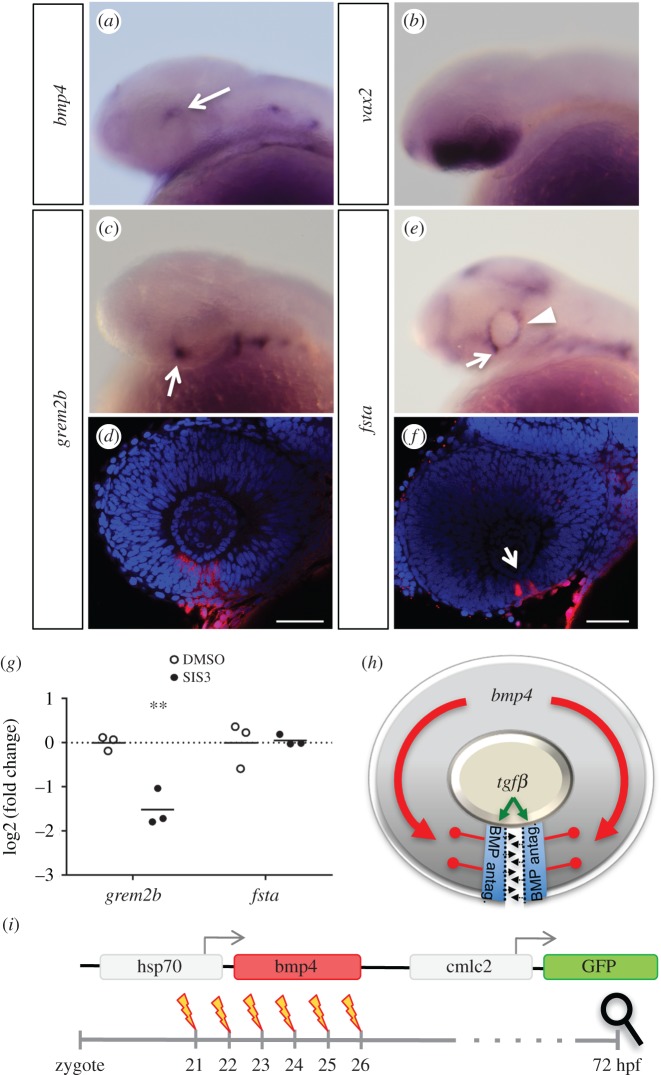
BMP antagonists *grem2b* and *fsta* are expressed in the optic fissure. WMISHs were performed at 30 hpf and are shown in lateral view. (*a*) *bmp4* is expressed in the dorsal optic cup (arrow). (*b*) Expression of *vax2* in the ventral retina. (*c*,*d*) Expression of *grem2b* in the optic cup is restricted to the optic fissure (arrow). (*d*) Confocal view of *grem2b* expression (red) with DAPI counterstaining (blue). (*e*,*f*) *fsta* is expressed in the optic fissure (arrows), as well as the ciliary marginal zone (CMZ, arrowhead). (*f*) Confocal view of *fsta* expression (red) with DAPI counterstaining (blue). (*g*) Expression analysis of gremlin and follistatin by quantitative PCR, differential expression in heads of SIS3-treated embryos (30 hpf) as represented by the log2(fold change) of individual samples. Embryos were treated from 24 hpf onward, controls were treated with DMSO. Material from three individuals was pooled for one sample; *n* = 3, horizontal bars represent the arithmetic mean. *p*-Values for *grem2b* and *fsta*, 4.3 × 10^−3^ and 0.877, respectively. (*h*) Model of the proposed role of TGFβ and BMP antagonism during optic fissure fusion. TGFβ signalling domains in the optic fissure margins are shielded from BMP by induced BMP antagonists. (*i*) Scheme of a heat shock inducible BMP construct used to create the transgenic line *tg(hsp70:bmp4, cmlc2:GFP)*. GFP expressed under the cardiac *cmlc2* promoter serves as transgenesis marker. Experimental procedure using heat shocks at different time points between 21 and 26 hpf to induce *bmp4* expression, aiming at disrupting optic fissure fusion. Analysis of phenotypes was scheduled for 3 dpf.

We found two antagonists for BMP signalling, follistatin and gremlin1, transcriptionally downregulated in our murine coloboma model (figure 1*e*). BMP4 in combination with Vax2 is important for the definition of cellular identities along the dorsal ventral axis within the vertebrate eye [47–50]. In line with these data we found *bmp4* expressed dorsally and *vax2* expressed ventrally within the zebrafish optic cup (figure 3*a*,*b*). Furthermore, we found homologous genes to the identified BMP antagonists (*grem2b* and *fsta*) expressed in the optic fissure margins of zebrafish also opposing the *bmp4* expression domain (figure 3*c*–*f*). The expression of *grem2b* also matched the expression pattern previously reported for the gene [51]. We also tested whether these antagonists are regulated by TGFβ in fish by comparing their expression in SIS3-treated embryos at 30 hpf with DMSO-treated controls using quantitative PCR. Inhibition of Smad3 signalling caused downregulation of *grem2b*, but not *fsta* (figure 3*g*). However, because we extracted RNA from embryonic heads and *fsta* is also expressed in the ciliary marginal zone and some parts of the brain, it would be plausible that a possible regulation by TGFβ exclusively in the optic fissure would not be detectable. Alternatively, it is also possible that *fsta* is not regulated by Smad3 or TGFβ at all in zebrafish.

**Figure 4.**
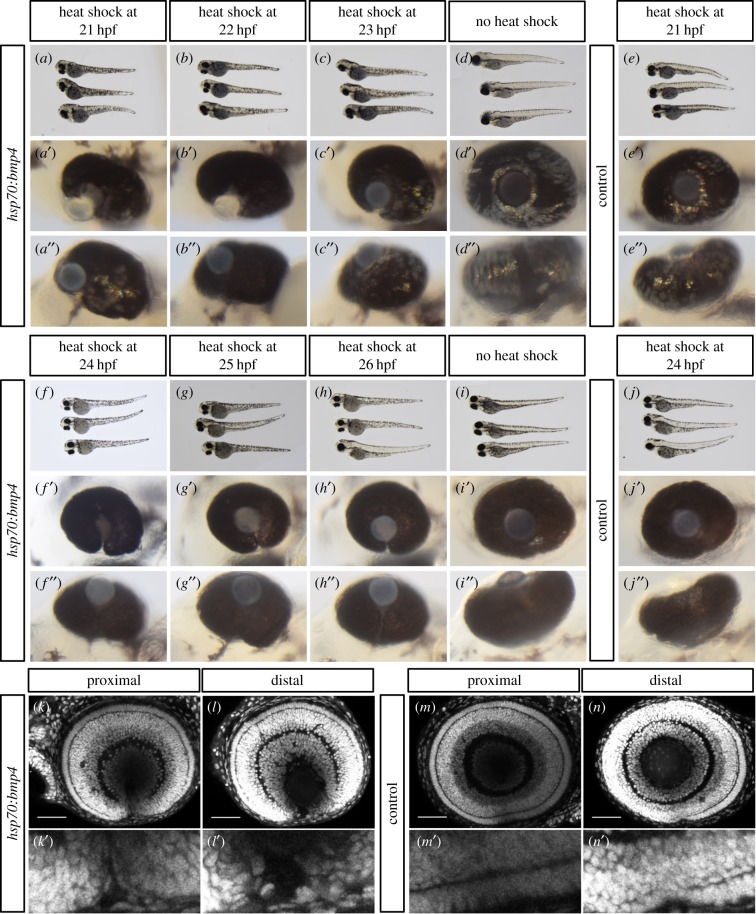
Differentially timed induction of *bmp4* causes different coloboma phenotypes. (*a*–*e*) Gross morphology of (*a*–*d*) *tg(hsp70:bmp4, cmlc2:GFP)* embryos after heat shock at 21, 22, 23 hpf, no heat shock, and (*e*) wild-type embryos heat shocked at 21 hpf. (*a*′–*h*′) Close up, lateral view, (*a*″–*h*″) close up, ventral views. Early *bmp4* induction impairs optic cup morphogenesis, resulting in coloboma (*a*′–*c*″). (*f*–*j*) Gross morphology of (*f*–*i*) *tg(hsp70:bmp4, cmlc2:GFP)* embryos after heat shock at 24, 25, 26 hpf, no heat shock and (*j*) wild-type embryos heat shocked at 24 hpf. (*f*′–*j*′) Close up, lateral view, (*f*″–*j*″**)** close up, ventral views. Late *bmp4* expression hampers optic fissure fusion, resulting in coloboma (*f*′–*h*″). All animals were visualized at 3 to 3.5 dpf. (*k*–*n*) Lateral confocal images of larval eyes (3 dpf) stained with DAPI. Individuals were heat shocked at 24 hpf. *Tg(hsp70:bmp4, cmlc2:GFP)* animals show a persisting optic fissure (*k*,*l*) while their wild-type siblings do not (*m*,*n*). (*k*′–*n*′) are magnifications of the optic fissure region. Notably, the fissure margins are touching in the proximal part of the eye (*k*′).

BMP4 is a secreted ligand and can potentially diffuse and act over extended distances. The expression of the two BMP antagonists in the optic fissure hints at a functional requirement to locally suppress BMP activity in this domain. Our data indicate that TGFβ signalling is relevant for ECM remodelling during optic fissure fusion. Notably, BMP signalling was shown to potentially counteract such TGFβ-induced changes [29–32]. We propose that TGFβ-induced local BMP antagonism is protecting the expression of TGFβ-regulated genes, which are facilitating ECM remodelling during optic fissure fusion (figure 3*h*, scheme). We next wanted to functionally test our hypothesis. We generated a transgenic line allowing heat shock inducible *bmp4* expression (*tg(hsp:bmp4 cmlc2:GFP)*) (figure 3*i*). With this heat shock inducible transgenic line, we aimed at an oversaturation of the BMP antagonists within the optic fissure margins. Since the morphogenesis of the optic cup is dependent on BMP antagonism [11], the timing of the heat shock induced expression of *bmp4* was critical. Thus, we tested the outcome of the heat shock induced *bmp4* expression at different successive stages of development (figure 3*i*). Induced expression at 21 hpf and 22 hpf resulted in an extended coloboma (figure 4*a*–*a*″ and *b*–*b*″, see *d*–*e*″ as control), well in line with the coloboma observed in our previous analyses [11]. This indicates that the transgenic line is functional and sufficiently high BMP4 levels are expressed but it also indicates that the onset of induction was too early and was affecting optic cup morphogenesis. The induced expression of *bmp4* at 23 hpf resulted in a milder coloboma, with less affected cup morphogenesis (figure 4*c*–*c*″). Notably, the coloboma phenotypes resulting from *bmp4* expression induced at 24, 25 and 26 hpf were comparable. Importantly, they were not showing defects in optic cup morphogenesis (figure 4*f*–*h*″, see *i*–*j*″ as control, and figure 4*k*–*n*). In the proximal part of the optic cup, the optic fissure margins were closely aligned but not fused (figure 4*k*′). Thus, we identified this phenotype as a defect in optic fissure fusion.

**Figure 5.**
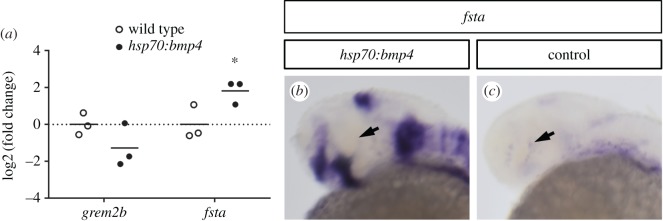
Timed induction of *bmp4* affects expression of BMP antagonists. (*a*) Expression analysis of gremlin and follistatin by quantitative PCR, differential expression in heads of *tg(hsp70:bmp4, cmlc2:GFP)* embryos at 30 hpf after heat shock at 24 hpf as represented by the log2(fold change) of individual samples. Material from three individuals was pooled for one sample; *n* = 3, horizontal bars represent the arithmetic mean. *p*-Values for *grem2b* and *fsta*, 0.170 and 0.049, respectively. (*b*,*c*) WMISH for *fsta* (30 hpf) in *tg(hsp70:bmp4, cmlc2:GFP)* after heat shock at 24 hpf (*b*) and control embryos (*c*). Strong upregulation after the heat shock is seen in the optic fissure and other expression domains, but not the ciliary marginal zone (arrow). WMISHs in (*b*) and (*c*) were stained in parallel for the same amount of time.

Next, we wanted to know whether the heat shock induced overexpression of *bmp4* affected the downstream signalling targets of TGFβ in the optic fissure. Therefore, we extracted RNA for quantitative analysis from the heads of 30 hpf *tg(hsp:bmp4 cmlc2:GFP)* embryos which had been subjected to a heat shock at 24 hpf. Wild-type siblings from the same clutch of eggs served as a control. We found that *grem2b* was downregulated in the *bmp4* overexpression condition by trend, although this result was not statistically significant (*p* = 0.17), while *fsta* was strongly upregulated (figure 5*a*). This upregulation was also seen in *in situ* hybridization of *fsta* and affected the expression domains in the trunk, the brain and the optic stalk and optic fissure, but not the ciliary marginal zone (figure 5*b*,*c*).

## 3. Discussion

Many genes and signalling pathways have been linked to coloboma [2–11] and a resulting coloboma gene network [12,13] is growing. The morphology of the coloboma phenotypes, however, is highly variable and many phenotypes likely result from early morphogenetic defects (e.g. [11]). On the other hand, little still is known about the process of optic fissure fusion. Recently, it was shown that hyaloid vessels are important for basement membrane degradation [22], a process important at the initiation of optic fissure fusion. This process is likely involving the transcription factors Pitx2 [52], Pax2 [14] and Vax2 [53] and potentially also retinoic acid signalling [52]. However, with respect to the subsequent process, the actual fusion of the optic fissure margins, less data is available.

It is unclear how the structure of the epithelial margins is loosened and eventually disassembled locally and how new connections are established, linking the neuroretinal domains and the RPE domains of both margins. Especially for the epithelial disassembly, the molecular mechanism is elusive. Concerning the formation of new connections, *n-cadherin* and *α-catenin* [20,21] are good candidate genes. The morphology of the coloboma resulting from a conditional *α-catenin* mutant shows an alignment of the margins [21], indeed indicating a functional involvement related to the fusion process. The coloboma resulting from *n-cadherin* mutation, however, shows a certain remaining gap [20], suggesting that not the process of fusion but a preceding event was affected by the mutation.

Our data indicate that TGFβ signalling is necessary for the fusion of the optic fissure margins. Hereby, we are integrating TGFβ as a new member into the coloboma gene network. Besides, TGFβ signalling is also known to be essential for palatal fusion [33,54]. Notably, the ligands TGFβ2 and TGFβ3 have slightly different functions there. While in TGFβ2 mutant mice the palatal shelves stay apart and a gap remains [33], in TGFβ3 mutants the palatal shelves potentially touch, but do not fuse [54,55]. The latter is reminiscent of what we observed in our study: the fissure margins meet, but do not fuse (figure 1*b*).

During our analysis we observed a potential influence of the genetic breeding background on optic fissure closure. Firstly, optic fissure fusion seemed to occur one day earlier in wild-type embryos from the sole breeding background (around E13) compared to those from the mixed breeding background (around E14). Secondly, the marked, strong coloboma phenotypes can be observed in both TGFβ2^−/−^ GDNF^−/−^ and TGFβ2^−/−^ GDNF^+/+^ embryos from a mixed breeding background, occasionally also presenting a dorsal coloboma, while the phenotype of TGFβ2 KO embryos from a sole breeding background is noticeably milder (figure 1*a*–*d*, electronic supplementary material, S1B–C). Lastly, in gene expression analysis, we found significant downregulation of Grem1 and Fst in TGFβ2^−/−^ GDNF^+/−^ eyes compared to TGFβ2^+/+^ GDNF^+/−^ eyes (figure 1*f*), whereas in the TGFβ2 KO derived from a sole breeding background, Grem1 was not significantly regulated and Fst downregulation was less pronounced (figure 1*i*). We want to mention that these effects could also be due to or maybe just influenced by a yet unreported sensitizing effect of GDNF signalling. Even if GDNF single KO embryos do not show a coloboma and the transcriptional analyses performed during our analyses contradict, we cannot totally rule out that an affected GDNF signalling sensitizes the system to the loss of TGFβ2. An interaction between these two TGFβ subfamilies (TGFβ and GDNF) is well known [56–58]. However, the signalling interaction in such scenarios is occurring vice versa, and TGFβ was found to facilitate GDNF signalling. Overall it seems more likely that the documented ocular GDNF expression [38] is important for later stages of development, because GDNF was previously reported to promote the proliferation and differentiation of photoreceptors during eye development [59]. It is expressed in the lens and retina of E14.5 mouse embryos [38], which is after the closure of the optic fissure, during differentiation of the neuroretina. Taken together, we consider it more likely that the differences we observe in phenotypes and transcriptomes are due to the genetic background of the mouse strains used, although we cannot rule out a contribution of GDNF based on our data.

TGFβ signalling is well-described to be an important regulator of modifications to the ECM in development and disease [23–28]. TGFβ-induced changes to the ECM are, however, potentially counteracted by BMP signalling [29–32]. BMP signalling and BMP antagonism are crucial during many important steps of eye development, such as optic fissure generation [60], dorsal–ventral cell specification and optic cup formation [11]. In the latter study, we showed that an inhibition of BMP signalling is crucial for a bilateral neuroretinal flow to be maintained over the distal rim of the developing optic cup. This BMP signalling inhibition was achieved by the BMP antagonist follistatin a (*fsta*). Considering also the early expression domains of *grem2b* [51] it seems likely that these two BMP antagonists also cooperate during optic cup formation during neuroretinal flow maintenance.

Here, we propose that TGFβ-induced local BMP antagonism in the optic fissure is protecting the expression of TGFβ-regulated genes which facilitate fissure fusion. A similar process, involving the BMP antagonist gremlin, was observed in the context of glaucoma [61]. Gremlin has, furthermore, been linked to cleft lips in humans [62], indicating that the level of BMP signalling must be tightly controlled during fusion processes there as well. A locally induced BMP antagonism within the fissure margins seems especially important because also other BMP ligands besides *bmp4* are expressed within the optic cup, e.g. *bmp2b*, *bmp2a* and *bmp6* and *bmp7b* [63–66]. The combined expression of two BMP antagonists within the optic fissure margins is likely important to provide robustness to the system. BMP antagonists are often expressed redundantly [67–69], pointing at the importance of their function. Thus, it is not surprising that the loss of a single BMP antagonist does not result in coloboma (Grem1 mutant mice). In TGFβ2 KO embryos with a sole breeding background, we found that Grem1 was noticeably less downregulated compared to embryos with a mixed breeding background, while Fst downregulation was changed only slightly. This might explain the less severe phenotype that we observed in the colobomatous embryos from a sole breeding background (figure 1*d*).

While our data strongly indicate that BMP antagonism is important for optic fissure fusion, we do not yet understand how BMP signalling can interfere with this process, specifically how it could counteract the changes induced by TGFβ signalling. BMP might repress the expression of TGFβ receptors or signal transduction components directly. In mouse pulmonary fibroblasts, BMP was shown to reduce TGFβ-dependent collagen expression by inducing inhibitors of differentiation 2 and 3 (Id2/3) [29]. The same study reported that nuclear localization of Smad3 in response to TGFβ signalling was inhibited by BMP, while another study found that Smad1 and Smad2/3 co-localized in nuclei of renal tubules in response to simultaneous TGFβ and BMP signalling [30]. The inhibitory Smad7 [70] could potentially also be involved in the signalling interaction. In mouse, knockout of the Smad7 gene led to various eye defects including coloboma [71]. Thus, it appears that there is a spectrum of TGFβ–BMP interaction which depends on the biological context and may include transcription-dependent and -independent mechanisms. Further research is needed to determine the mode of interaction between the two pathways in the context of tissue fusion and whether BMP signalling is directly inhibiting the TGFβ signalling pathway or only downstream targets of TGFβ. Our results suggest that only a subset of TGFβ target genes might be inversely regulated by BMP signalling but that these genes would be crucial for the optic fissure fusion process.

Our data obtained by timed overexpression of *bmp4* in zebrafish embryos clearly indicate that BMP induction has a detrimental effect on optic fissure fusion, resulting in coloboma (figure 4). We showed, in accordance with our previous findings [11], that *bmp4* overexpression during optic cup formation (prior to 24 hpf) causes morphogenetic defects by disrupting epithelial flow. Only overexpression after 24 hpf was able to induce a defect of optic fissure fusion. Under this condition, we also observed a repression of the TGFβ target gene *grem2b* by trend (figure 5*a*). However, this result was not statistically significant, thus leaving the relevance unclear. By contrast and unexpectedly, *fsta* was found upregulated after forced *bmp4* expression (figure 5). This could be a response of the developing organism to the increased levels of BMP, trying to reduce BMP signalling to a physiological level by upregulating *fsta*. Notably, the fissure margins were responding intensively in comparison to the ciliary marginal zone, where hardly any change of expression was noticeable. Furthermore, *fsta* was not found downregulated in response to SIS3 treatment (figure 3*g*). This could imply that *fsta* is not regulated by Smad3, at least in zebrafish. It is however still possible that it is instead regulated through Smad2. Several genes are known that possess binding sites for only one of these two Smads, including Goosecoid (*gsc*) and *mix2* for Smad2, as well as JunB/*junba* and PAI-1/*serpine* for Smad3 [41,72–74]. Another interpretation would be that *fsta* is not regulated by TGFβ in zebrafish.

It should be mentioned that a completely different interpretation is also supported by our data, in which the upregulation of *fsta* is not a compensatory effect but the cause for the optic fissure closure defect that we observe after BMP overexpression. In that case, the mechanism would be independent of TGFβ. While we cannot exclude this option, we consider it unlikely because *fsta* is downregulated in the TGFβ2 KO mouse model. In summary, we conclude that *fsta* is either not controlled by Smad3 or TGFβ in zebrafish, or that due to differential regulation of the expression domains in the fissure margins and the ciliary marginal zone, a subtle regulation within only the margin domain was not detectable by qPCR.

## 4. Conclusion

The process of optic fissure fusion is not well understood, especially not on a cellular and molecular basis. We found an interplay of two growth factors, TGFβ and BMP, during the fusion of the optic fissure margins. While TGFβ signalling is acting pro-fusion and induces changes to the ECM, we found that BMP signalling is capable of inhibiting fissure fusion. Notably, TGFβ signalling is inducing BMP antagonists within the fissure margins. This finding suggests that thereby TGFβ, itself acting pro-fusion, is also locally counteracting BMP signalling, which is acting anti-fusion. Even if gene regulation might differ depending on the breeding background and between fish and mouse, the functional concept appears to be conserved. Our findings can likely be applied also to other fusion processes, especially when TGFβ signalling or BMP antagonism is involved, as in fusion processes during orofacial development. Together with our previous data, our current work indicates a dual role of BMP antagonism, first during optic cup formation, maintaining a bilateral neuroretinal flow entering the optic cup, and second during optic fissure fusion.

## 5. Material and methods

### 5.1. Mice

For this study TGFβ2^+/−^ [33] and GDNF^+/−^ [35] mice were used for breeding. Timed matings were performed overnight and the day on which a vaginal plug was visible in the morning was considered as day 0.5. Analyses were restricted to embryonic stages because of perinatal lethality of the individual mutants. For analysis of embryonic tissue, the mother was sacrificed and the embryos were collected by caesarean section. All of the experiments were performed in agreement with the ethical committees. Genotyping was performed according to Rahhal *et al.* [34]. The term ‘TGFβ2 KO with mixed breeding background' refers to a TGFβ2^−/−^, GDNF^+/+^ offspring of TGFβ2^+/−^, GDNF^+/−^ parents, because the original single mutant lines were created in different wild-type strains [33,35]. A ‘sole breeding background' refers to the TGFβ2 line [33]. This line was maintained and crossed with C57BL/6.

### 5.2. Histological analysis

Tissue was processed for paraffin sectioning. Frontal sections of control and TGFβ2^−/−^ embryos were obtained and stained with haematoxylin and eosin.

### 5.3. Microarray data

RNA was extracted from whole eyes of E13.5 embryos (controls and TGFβ2^−/−^ (GDNF^−/−^) respectively). RNA was reverse transcribed, amplified and loaded on Agilent one-colour microarray chips. Experiments were performed in triplicates.

Further analysis was performed using R [75] and the bioconductor packages Agi4×44PreProcess, limma and mgug4122a.db as annotation database. For background correction we used the following parameters: BGmethod = ‘half', NORM-method = ‘quantile', foreground = ‘MeanSignal’, background = ‘BGMedianSignal' and offset = 50. The probes were filtered using the recommended thresholds and afterwards the replicated non-control probes were summarized. Then the method *lmFit* was used to fit a linear model on the arrays. Finally, the differential expression statistics were computed using the methods *eBayes*.

Next only those genes with fold change higher than 1.5 were considered, then a multiple comparison correction was performed on the *p*-values using the BH (Benjamini and Hochberg) method. The genes with corrected *p*-value lower than 0.05 were defined as significantly differentially expressed genes. The microarray data supporting this article have been made available via the repository ‘BioStudies’ under the identifier S-BSST80.

### 5.4. Functional analysis of gene sets

We used the tool gProfiler ([76], http://biit.cs.ut.ee/gprofiler/) version 6.7 to find enriched terms on the set of significantly downregulated genes from the mouse arrays. We provided the official gene symbol of these genes as input and used the default set of databases.

### 5.5. RNA extraction from mouse and zebrafish tissue for quantitative PCR

RNA was extracted from whole eyes of E12.5 embryos (controls and TGFβ2^−/−^, respectively). For zebrafish, three heads from 30 hpf embryos were pooled for each sample. RNA was extracted using the guanidinium thiocyanate–phenol–chloroform method (modified after [77]). After DNaseI treatment, it was purified again by phenol–chloroform extraction and sodium acetate precipitation.

### 5.6. Quantitative PCR

RNA was reverse transcribed with the ProtoScript II First Strand cDNA Kit (New England Biolabs). Quantitative realtime PCR was performed with a CFX Connect Real-Time PCR Detection system (Bio-Rad Laboratories) and the Luna Universal qPCR Master Mix (New England Biolabs) in technical triplicates with 20 µl reaction volume and 2 µl of a 1:10 dilution of the cDNA template. *Gapdh* was used as reference gene for mouse qPCR and *eef1a1l* was used for zebrafish qPCR. For primers used, see electronic supplementary material Information.

For data analysis, the dCq values were calculated by subtracting Cq[_target gene_] from Cq[_reference gene_], and ddCq values were calculated by subtracting the averaged dCq[_control_] from the simple dCq[_treatment/KO_] [78]. These ddCq values are presented as log2(fold change). *p*-Values were determined by two-tailed, unpaired *t*-test, except for the data presented in figure 1*f*. There we used a one-tailed test because we knew to expect downregulation from the microarray dataset.

In figure 5*a*, the expression data for *grem2b* derives from two runs with the same samples (biological replicates) in the same qPCR cycler. For this, an average dCq value was calculated for each biological replicate.

### 5.7. Zebrafish husbandry

Zebrafish (*D. rerio*) were kept as closed stocks in accordance with local animal welfare law and with the permit 35-9185.64/1.1 from the Regierungspräsidium Freiburg. The fish were maintained in a constant recirculating system at 28°C on a 12 L : 12 D cycle. Fish lines used in this study were created in the AB wild-type strain.

### 5.8. Transgenic zebrafish

Plasmid DNA containing SBEs in combination with a minimal promoter were kindly provided by Peter tenDijke ((CAGA)_12_ MLP Luc). Here, repetitive SBEs derived from the promoter of the human PAI gene [41] were used to drive a luciferase gene.

We cloned the SBEs with the minimal promoter into a Gateway 5′ entry vector (Invitrogen). A multisite Gateway reaction [79] was subsequently performed resulting in an SBE driven GFPcaax construct (SBE:GFPcaax). A zebrafish line was generated with SB (sleeping beauty) transgenesis according to Kirchmaier *et al.* [80]. Shh reporter zebrafish were generated according to Schwend *et al.* [45]. The plasmid was kindly provided by Sara Ahlgren.

We assembled the expression construct for *Tg(hsp70:bmp4, cmlc2:eGFP)* in a Gateway reaction, using a Tol2 destination vector including *cmlc2:eGFP* [79], a 5′entry vector containing the *hsp70* promotor, a pENTR D-TOPO (ThermoFisher Scientific) vector containing the CDS of *bmp4* [11] and a 3′entry vector with a polyadenylation site [79]. The construct (10 ng µl^−1^) was injected into wild-type zebrafish zygotes together with Tol2 transposase mRNA (7 ng µl^−1^) [79]. Embryos with strong GFP expression in the heart were selected as founders. Lines were kept in closed stocks and validated in every generation.

### 5.9. Drug treatments

Zebrafish embryos were treated with SIS3 (9 µM, 3 mM stock in DMSO [81]) and with SB431542 (80 µM, 10 mM stock in DMSO [82]). Controls were treated with DMSO without the inhibitor.

### 5.10. Microscopy

Signalling reporter fish were imaged with a Leica SP5 setup, samples were mounted in glass bottom dishes (MaTek). For time-lapse imaging embryos were embedded in 1% low melting agarose covered with zebrafish medium and anaesthetized with tricaine. Left and right eyes were used and oriented to fit the standard views. A stereomicroscope (Olympus/Nikon) was used for recording bright field and fluorescent images of TGFβ signalling reporter fish. Whole-mount *in situ* hybridizations were recorded with a stereomicroscope (Nikon SMZ18) as well as an upright microscope (Zeiss) and a confocal Leica SP8 setup.

### 5.11. Heat shocks and controls

*tg(hsp70:bmp4, cmlc2:eGFP)* eggs were kept in a Petri dish at 28°C after fertilization. To induce *bmp4* expression, 21–26 hpf embryos were transferred to a 1.5 ml reaction tube and incubated for 1 h at 37°C in a heating block. Afterwards, they were returned to a dish at 28°C. Embryos were fixed with 4% PFA at 30 hpf for *in situ* hybridization and at 3 dpf for morphological analysis.

We used *tg(hsp70:bmp4, cmlc2:eGFP)* embryos which were not heat shocked as controls, as well as heat shocked wild-type siblings from the same clutch of eggs.

### 5.12. Whole-mount *in situ* hybridization

Whole-mount *in situ* hybridizations (WMISHs) were performed according to Quiring *et al*. [83]. WMISHs for confocal imaging were stained with FastRed Naphthol (Sigma-Aldrich).

### 5.13. Immunohistochemistry and confocal imaging of zebrafish embryos

Fixed embryos were bleached using 3% hydrogen peroxide and 0.5% potassium hydroxide. For whole-mount imaging, they were stained with 4 µg ml^−1^ 4′,6-diamidino-2-phenylindole (DAPI) for 2 h. Imaging was performed in glass bottom dishes (Matek) using a Leica SP8 TCS setup.

For anti-Laminin staining, embryos were cryosectioned (20 µm) and stained on Superfrost plus slides. Antibodies used: rabbit Laminin Ab-1 (ThermoFisher Scientific, RB-082-A1, 1 : 100), goat anti-rabbit-Alexa 555 (ThermoFisher Scientific, A-21428, 1 : 250).

## Supplementary Material

Figure S1: Supplemental images of TGFβ2 KO coloboma phenotypes

## Supplementary Material

Figure S2: Establishment of a TGF signalling reporter in zebrafish

## Data accessibility

The datasets supporting this article have been uploaded as part of the supplementary material. The microarray data supporting this article have been made available via the repository ‘BioStudies’ under the identifier S-BSST80.
