# Supplementary figures and images for: TGFβ-facilitated optic fissure fusion and the role of bone morphogenetic protein antagonism
Source: Open Biol. 2018 Mar 28;8(3):170134. doi: 10.1098/rsob.170134 (PMC5881030; doi:10.1098/rsob.170134)

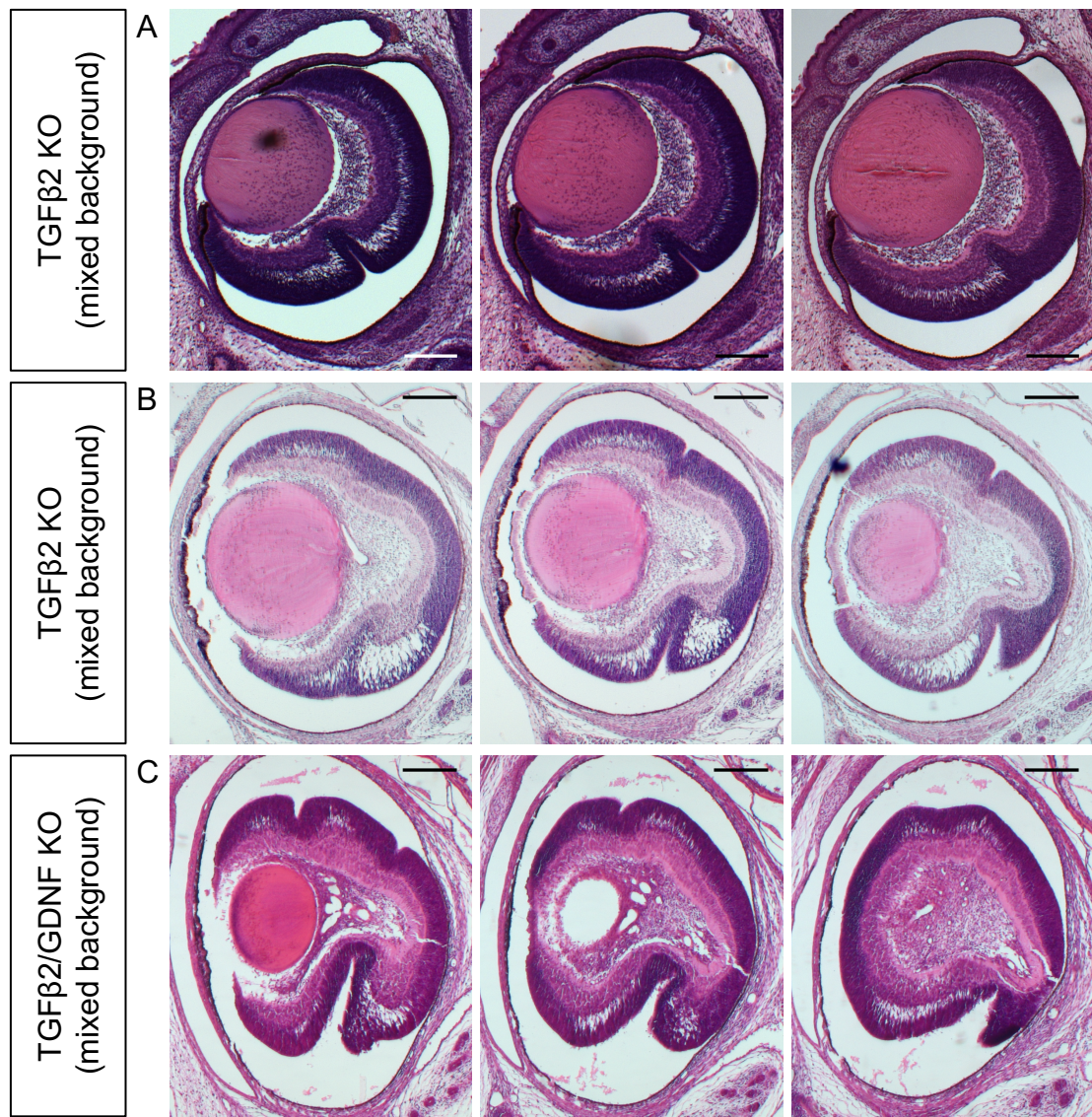

Figure S1 Knickmeyer et al., 2017

Supplement: Figure S1: Supplemental images of TGFβ2 KO coloboma phenotypes [file rsob170134supp1.pdf]

A

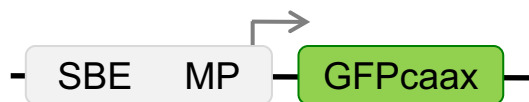

B

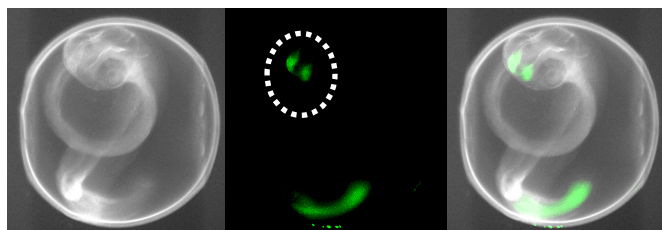

C

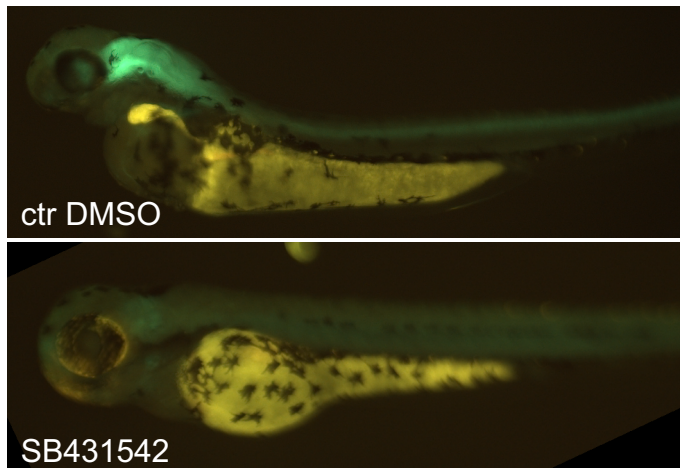

Supplement: Figure S2: Establishment of a TGF signalling reporter in zebrafish [file rsob170134supp2.pdf]
